# The effect of a low renal acid load diet on blood pressure, lipid profile, and blood glucose indices in patients with type 2 diabetes: a randomized clinical trial

**DOI:** 10.1186/s12937-023-00849-6

**Published:** 2023-03-15

**Authors:** Maryam Armin, Zahra Heidari, Gholamreza Askari, Bijan Iraj, Cain C. T. Clark, Mohammad Hossein Rouhani

**Affiliations:** 1 Nutrition and Food Security Research Center and Department of Community Nutrition, School of Nutrition and Food Science, Isfahan University of Medical Sciences, Isfahan, Iran; 2 Department of Biostatistics and Epidemiology, School of Health, Isfahan University of Medical Sciences, Isfahan, Iran; 3 Isfahan Endocrine and Metabolism Research Center, Isfahan University of Medical Sciences, Isfahan, Iran; 4 Centre for Intelligent Healthcare, Coventry University, CV1 5FB Coventry, UK

**Keywords:** Type 2 diabetes, Dietary renal acid load, Blood pressure, Lipid profiles, Glycemic control, Insulin resistance

## Abstract

**Background:**

Observational studies have reported that dietary renal acid load has an important role in insulin resistance and metabolic factors. The aim of the present study was to assess the effect of a low renal acid load diet (LRALD) on blood pressure, lipid profile, and blood glucose indices in patients with type 2 diabetes.

**Methods:**

In this parallel randomized clinical trial, 80 patients with type 2 diabetes were randomly assigned to the LRALD (*n* = 40) or control (*n* = 40) groups, for 12 weeks. Both groups received a balanced diet and a list of nutritional recommendations based on healthy eating behaviors. In the LRALD group, food items with low renal acid load were prescribed. Primary outcomes including: fasting blood glucose (FBG), hemoglobin A1c (HbA1c), fasting serum insulin, quantitative insulin sensitivity check index (QUICKI), homeostatic model assessment for insulin resistance (HOMA) and secondary outcomes including: weight, systolic blood pressure (SBP), diastolic blood pressure (DBP), triglyceride (TG), total cholesterol (TC), low-density lipoprotein (LDL), and high-density lipoprotein (HDL). were measured at baseline and end of the study. The present trial was registered at IRCT.ir (IRCT20130903014551N5).

**Results:**

Seventy subjects completed the study (*n* = 35 in control group and *n* = 36 in LRALD). Weight (*P* < 0.001), body mass index (*P* < 0.001), FBG (*P* < 0.001), HbA1c (*P* < 0.001), SBP (*P* = 0.004), and TG (*P* = 0.049) were reduced and HDL (*P* = 0.002) was increased in both groups, compared with baseline. After adjusting for baseline values, DBP (*P* = 0.047) was reduced in the LRALD group compared with control group. Results had no changes after using intention to treat analysis.

**Conclusion:**

A LRALD may decrease DBP in type 2 diabetic patients. However, it elicited no significant effect on lipid profile compared with a healthy diet.

**Trial registration:**

This randomized clinical trial was registered at IRCT.ir (IRCT20130903014551N5).

**Supplementary Information:**

The online version contains supplementary material available at 10.1186/s12937-023-00849-6.

## Introduction

Type 2 diabetes is one of the most prevalent non-communicable metabolic disorders, that results in a high rate of morbidity and mortality, worldwide [1], and, by 2030, the number of people with type 2 diabetes is estimated to exceed 552 million [2]. Uncontrolled type 2 diabetes may lead to retinopathy, nephropathy, heart diseases, stroke, and reduced life expectancy [2], whilst lifestyle modification, including dietary intervention, has an important role in management of type 2 diabetes [3].

Dietary intake is a determinant of acid production and may influence on acid–base balance in the body [4]; indeed, foods rich in components metabolized to acid precursors (i.e., sulfur and cationic amino acids including cysteine, methionine, taurine, lysine and arginine) may increase acid production [5]. In contrast, potassium, magnesium, and calcium are considered as alkali nutrients [5]. Since acid producing foods, including animal proteins and processed foods, were rarely consumed before the industrial revolution, it seems that humans may not have adapted to the contemporary acid producing dietary pattern, which may be a contributing factor to the current epidemics of chronic diseases, such as type 2 diabetes and obesity [6].

One of the main indicators used to estimate the acid renal load is potential renal acid load (PRAL), which refers to the intestinal absorption of five nutrients, including protein, potassium, calcium, phosphorus, and magnesium. A positive PRAL indicates acid-inducing and a negative score indicates alkali-inducing properties [7]. Net endogenous acid production (NEAP) is another index of dietary renal acid load that indicates the ratio of protein to potassium content of foods [8].

Several observational studies have been conducted to investigate the relationship between dietary acid load and glycemic control and metabolic indices. A prospective cohort study conducted on middle-aged subjects reported that a high acid-load diet was associated with a higher risk of type 2 diabetes [9]. A cross-sectional study conducted on participants aged 40 to 85 years revealed a positive relationship between dietary acid load and cardiovascular disease [10], whilst dietary renal acid load has been directly associated with gestational type 2 diabetes [11]. Furthermore, a meta-analysis, that pooled the results of the seven observational studies, reported that a high acid-load diet increased the risk of type 2 diabetes [12].

Although the number of observational studies that assessed the association between dietary acid load and glycemic control and metabolic factors are informative, no well-designed interventional study has been performed to detect the effect of dietary renal acid load on metabolic factors in type 2 diabetic patients. Therefore, in the present study, we aimed to evaluate the effects of a low renal acid load diet (LRALD) on, blood glucose, and insulin resistance as primary outcomes and anthropometric variables, blood pressure and lipid profiles as secondary outcomes in patients with type 2 diabetes.

## Subjects and methods

### Subjects

This parallel randomized clinical trial was conducted from June to September 2020. Adults with type 2 diabetes were recruited from a governmental type 2 diabetes center. Individuals were included if they were: 1) between 20 to 65 years old, 2) type 2 diabetic patients not on insulin therapy, 3) not pregnant or lactating, 4) not on glucocorticoids and 5) not underweight (body mass index (BMI) > 18.5 kg/m^2^). Subjects who started using a new blood glucose-lowering drug, changed the dose of medications, or had a diabetic ketoacidosis attack during the study were excluded. The number of subjects was calculated based on fasting blood glucose (FBG) by using following formula: *n* = 2 [(Z _(1-α/2)_ + Z_(1-β)_)^2^ × S^2^] / Δ^2^, where α = 0.05 (type I error), β = 0.20 (type II error), Δ = 15.63 mg/dl and S = 32.91 mg/d [13]. Therefore, the minimum required sample size for the present study was 70 (*n* = 35 in each group). Accordingly, we recruited 40 subjects in each group at baseline, to account for loss to attrition. The aims and details of the study were individually explained for each volunteer. All participants signed an informed written consent forms before study commencement. This study was approved by the Research Council and Ethical Committee of the School of Nutrition and Food Science, Isfahan University of Medical Sciences, Isfahan, Iran and the Food Security Research Center, Isfahan University of Medical Sciences, Isfahan, Iran. Present trial was registered at IRCT.ir (IRCT20130903014551N5).

### Study procedure and dietary intervention

Both groups were recommended a balanced diet modified for type 2 diabetic patients (such as carbohydrate counting) Energy intake was calculated for normal weight and overweight/obese subjects using current body weight and adjusted ideal body weight, respectively. Energy requirement was calculated using the Mifflin St Jeor Equation [14]. The diet in both groups contained 52–53% of carbohydrates, 17–18% of protein, and 30–31% of fat. In order to prevent ketosis, the carbohydrate content of the diets was above 130 g/d. Volunteers in both groups received a list of nutritional recommendations based on healthy eating behaviors including: 1) Meals should be small, frequent and used regularly, 2) Do not change carbohydrate content of your diet without consulting your dietician 3) Restrict intake of refined carbohydrate, Whole grains are preferable to refined grains, 4) Eat vegetables frequently, 5) Use fruits with skin if possible and 6) Fruits are preferable to fruit juice. Daily meal plans were designed according to the potential renal acid load (PRAL) of food items [15] only in LRALD group. Foods with high acid load (PRAL > 4), except chicken meat, were excluded from the diet of LRALD group. Chicken meat, one of the most frequently consumed foods, was limited to one serving per day. Also, two fixed snacks contained very low PRAL foods (foods with PRAL < -4 such as spinach, celery, squash, and raisins) were prescribed in LRALD group. A colored list of the food items was provided, in which the red color was used for foods with high PRAL and low-PRAL foods were demarcated by the color green. Subjects in the LRALD group were educated to select green items and limit red foods. Dietary recommendations for two group are reported in (Supplementary 1). Compliance with prescribed diets were assessed by food records. PRAL Table of foods are reported in (Supplementary 2). In the present study, dietary acid load indices were estimated using the following formulas [16]:

$$\mathrm{PRAL}\left(\mathrm{mEq}/\mathrm d\right)=0.4888\times\mathrm{protein\ intake}\left(\mathrm g/\mathrm d\right)+0.0366\times\mathrm{phosphorus}\left(\mathrm{mg}/\mathrm d\right)-0.0205\times\mathrm{potassium}\left(\mathrm{mg}/\mathrm d\right)-0.0125\times\mathrm{calcium}\left(\mathrm{mg}/\mathrm d\right)-0.0263\times\mathrm{magnesium}(\mathrm{mg}/\mathrm d),$$

and [17]

$$\mathrm{NEAP}\left(\mathrm{mEq}/\mathrm d\right)=\left[54.5\times\mathrm{protein\ intake}\left(\mathrm{mg}/\mathrm d\right)\;\div\;\mathrm{potassium\ intake}\;\left(\mathrm{mEq}/\mathrm d\right)\right]-10.2$$

### Confounding variables

Potential confounding variables in this study were physical activity and food intake.

### Dietary intakes and physical activity

Six, one-day (4 weekdays and 2 weekends), food diaries were completed by participants at baseline and during the study. The validity and precision of the dietary record is high and it is often considered as a reference method in validation studies. Nevertheless, we checked all dietary records to complete unclear and incomplete reports by each participant. Also, we excluded dietary records that reported < 800 or > 4200 kcal energy intake per day. We guided participants to how complete a food record by several images regarding portions sizes. Also, a completed food record was provided for each participant as a sample. We checked all dietary records to complete unclear and incomplete reports by each participant. Food diaries were converted to the macro/micronutrients by Nutritionist IV using the USDA database [18, 19].

Participants were asked to complete 4 daily physical activity records during the study. Physical activity was calculated using Metabolic Equivalent per hour per day (MET.hd) [19]. Patients invited to meetings scheduled at baseline and 3-, 7- and 10 weeks to assess compliance. In addition, the subjects were individually monitored every week.

### Anthropometric measurements and blood pressure

Body weight was measured at the baseline and on the 12^th^ week, where subjects wore lightweight clothes and were unshod, using a standard scale, to the nearest 0.1 kg. Height was measured at baseline, using a wall-mounted stadiometer, with participants standing upright and unshod. Seated blood pressure was measured using a mercury sphygmomanometer, after 10 min of rest. The systolic blood pressure (SBP) and diastolic blood pressure (DBP) were recorded by the first sound and the fade of the sound, respectively.

### Biochemical measurements

In the early morning, a fasting (12 h) blood sample was drawn. Serum was separated by centrifuging at 3,000 × g for 10 min. Enzymatic colorimetric method was performed to measure FBG, serum concentration of triglyceride (TG) and total cholesterol (TC) (Pars Azmoon, Tehran, Iran). Similarly, high-density lipoprotein (HDL) and low-density lipoprotein (LDL) were measured after blocking other cholesterol containing components by photometric methods. Glycosylated hemoglobin (HbA1c) was assessed using ion exchange chromatography method. Fasting serum insulin was measured by enzyme-linked immunosorbent assay (ELISA) (Monobind Inc,Costa Mesa, CA, USA). To estimate insulin resistance, Quantitative insulin sensitivity check index (QUICKI) and homeostatic Model Assessment for Insulin Resistance (HOMA) were calculated by following formulas [20]:

$${\mathrm{HOMA}}= ({\mathrm{fasting\ glucose\ }}[{\mathrm{mmol}/\mathrm{L}}] \times {\mathrm{fasting\ insulin\ }}[{\mathrm{\mu U}/\mathrm{mL}}])/22.5$$

$$\mathrm{QUICKI }=1/ (\mathrm{log\ fasting\ glucose\ }[\mathrm{mg}/\mathrm{dL}] +\mathrm{ log\ fasting\ insulin\ }[\mathrm{\mu U}/\mathrm{mL}])$$

### Confounder variables

We considered age, sex, body mass index and physical activity as potential confounding variables. Previous studies revealed that age was related to the lipid profile [21] and blood glucose indices [22]. Also, serum lipids and glucose hemostasis may be affected by gender [23, 24]. Obesity is considered as a risk factor for high blood glucose [25] and abnormal blood lipids [26]. Evidence showed that physical activity may improve glucose hemostasis [27] and lipid profile [28].

### Statistical analysis

Normality of the data was checked using the Kolmogorov–Smirnov test and Q-Q plot, and results showed that the distribution of HOMA-IR, QUICKI, and TG was not normal. Therefore, we used the log transformed version of these variables. Chi-square tests were used to compare qualitative variables between the LRALD and control groups. Quantitative variables were reported as percentages. To compare baseline and endpoint values within each group, Paired T test analysis was used. Quantitative variables were compared between two groups using Independent Student t-test. To adjust for confounding variables (energy intake and baseline values), analysis of covariance (ANCOVA) was applied. To report primary and secondary outcomes, both per-protocol and intention to treat (ITT) analysis were used. Missed data were treated according to linear regression method. Continuous data were reported as mean ± standard deviation. The log-transformed variables were reported as geometric mean ± standard deviation. All data analyses were conducted using SPSS version 21 statistical software, with an *a* priori alpha level of 0.05.

## Results

**Fig. 1:**
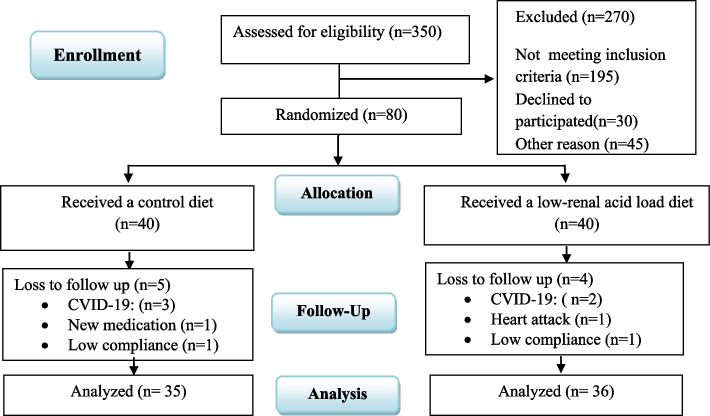
Flow Diagram illustrating participant selection process and study procedure

A flow diagram of the study procedure is illustrated in Fig. 1. Of 350 subjects were screened at baseline according to the inclusion and exclusion criteria, 80 patients who met these criteria and completed the informed written consent form. Then they were randomly assigned to the LRALD and control groups. During the study, 5 patients in the control group were excluded due to positive COVID-19 test (*n* = 3), taking a new drug (*n* = 1), and not following the prescribed diet correctly based on the patient's own confessions (*n* = 1). Four subjects were excluded from LRALD group due to positive COVID-19 test (*n* = 2), heart attack (*n* = 1), and low adherence to the prescribed diet based on the patient's own confessions (*n* = 1). Finally, data of 71 patients (*n* = 35 in control group and *n* = 36 in LRALD) were statistically analyzed. The number of the subjects who completed the study was greater than minimum required sample size reported in method section.

General characteristics of the subjects are reported in Table 1. There was no significant difference in age, sex, height, weight, BMI, and physical activity between two groups at baseline.

**Table 1:** Baseline characteristics of participants

Variable	LRALD group (*n* = 36)	Control group (*n* = 35)	*P* value ^a^
Age (y)	50.4 ± 10.5	47.6 ± 7.9	0.23
Male (%)	30.3	35.5	0.43
Height (m)	1.6 ± 0.1	1.6 ± 0.0	0.67
Weight (kg)	77.5 ± 15.6	73.8 ± 11.2	0.27
Body mass index (kg/m^2^)	28.7 ± 4.8	27.7 ± 3.7	0.33
Normal Weight (%)	18.2%	22.6%	0.48
Overweight (%)	45.5%	54.8%	
Obese (%)	36.4%	22.6%	
Physical Activity (MET hour/day)	33.4 ± 3.8	33.4 ± 2.4	0.98

All continuous variables are reported as Mean ± SD

*LRALD* Low renal acid load diet

^a^*P* values for continues and nominal variables were calculated by Independent t-test and Chi-square, respectively

Comparison of dietary intake during the study between LRALD and control groups is shown in Table 2. Results showed that intake of cholesterol, saturated fatty acid, polyunsaturated fatty acid, monounsaturated fatty acid, vitaminB3, calcium, magnesium, zinc, and phosphorus was significantly less in the LRALD group compared with the control group. In contrast, subjects in the LRALD group consumed more amounts of iron, potassium, beta-Carotene, folate, vitamin C, vitamin B1, vitamin E), vitamin A, and dietary fiber in comparison with control group. PRAL and NEAP, indicators of adherence to the LRALD, were significantly lower in the LRALD group compared with control group.

**Table 2:** Food intake during the study in low renal acid diet and control groups based on food records

Nutrients	LRAL diet (*n* = 36)	Control (*n* = 35)	*P* value ^b^
Energy (kcal)	1854 ± 342	1848 ± 249	0.94
Carbohydrate (g/d)	233^a^ ± 5	234^a^ ± 5	0.30
Protein (g/d)	83^a^ ± 5	83^a^ ± 5	0.62
Fat (g/d)	66^a^ ± 3	65^a^ ± 3	0.21
Cholesterol (mg/d)	103^a^ ± 122	398^a^ ± 122	< 0.001
SFA (g/d)	9.03^a^ ± 2	13^a^ ± 2	< 0.001
PUFA (g/d)	13^a^ ± 4	15^a^ ± 4	0.04
MUFA (g/d)	9^a^ ± 2	142^a^ ± 2	< 0.001
Dietary Fiber (g/d)	25^a^ ± 8	17^a^ ± 8	< 0.001
VitaminA (RE/d)	1132^a^ ± 427	481^a^ ± 427	< 0.001
VitaminE (mg/d)	19^a^ ± 5	14^a^ ± 5	< 0.001
VitaminC (mg/d)	86^a^ ± 24	28^a^ ± 24	< 0.001
VitaminB1 (mg/d)	2^a^ ± 0	2^a^ ± 0	< 0.001
VitaminB2 (mg/d)	2^a^ ± 0	2^a^ ± 0	0.84
VitaminB3 (mg/d)	20^a^ ± 3	22^a^ ± 3	0.01
VitaminB6 (mg/d)	1^a^ ± 1	1^a^ ± 1	0.14
Folate (µg/d)	478^a^ ± 120	246^a^ ± 120	< 0.001
Beta-Caroten (µg/d)	980^a^ ± 454	121^a^ ± 454	< 0.001
Potassium (mg/d)	3261^a^ ± 497	2844^a^ ± 497	0.001
Iron (mg/d)	16^a^ ± 1	13^a^ ± 1	< 0.001
Calcium (mg)	967^a^ ± 155	1095^a^ ± 155	0.001
Zinc (mg/d)	8^a^ ± 1	9^a^ ± 1	< 0.001
Magnesium (mg/d)	265^a^ ± 42	308^a^ ± 42	< 0.001
Phosphorus (mg)	1109^a^ ± 136	1202^a^ ± 136	0.008
Selenium (mg)	1^a^ ± 0	1^a^ ± 0	0.35
Chromium (mg)	1^a^ ± 2	1^a^ ± 2	0.34
PRAL(mEq/d)	-6.5 ± 11.5	3 ± 7	< 0.001
NEAP(mEq/d)	36.8 ± 14.1	45 ± 8	0.003

All variables are reported as Mean ± SD

*LRAL* Low renal acid load, *SFA* Saturated fatty acid, *PUFA* Polyunsaturated fatty acid, *MUFA* monounsaturated fatty acid, *PRAL* Potential renal acid load*, NEAP Net* endogenous acid production

^a^Values were adjusted for energy intake

^b^Calculated by ANCOVA except for energy, PRAL and NEAP calculated by independent t-test

As shown in Table 3, the results of comparing anthropometric indices, lipid profile, fasting blood sugar, and blood pressure in the LRALD and control groups before and after the study.

**Table 3:** Comparison of anthropometric indices, lipid profile, fasting blood sugar, and blood pressure in the low renal acid diet and control groups before and after the study

	***LRAL diet*** ***(n* = *36)***				***Control*** ***(n* = *35)***				***P***^***c***^	***P***^***d***^
	Baseline	End of trial	Change	P^b^	Baseline	End of trial	Change	P^b^		
**BMI (kg/m**^**2**^**)**	28.7 ± 4.8	27.3 ± 4.57	-1.4	< 0.001	27.7 ± 3.7	26.8 ± 3.6	-0.9	< 0.001	0.21	0.21
**Weight (kg)**	77.6 ± 15.6	74.5 ± 14.68	-3.0	< 0.001	73.8 ± 11.2	71.5 ± 10.1	-2.3	< 0.001	0.46	0.46
**FBG (mg/dl)**	192.3 ± 68.2	155.0 ± 51.45	-37.2	< 0.001	190.5 ± 78.2	160.1 ± 66.4	-30.5	0.01	0.59	0.59
**HbA1C (%)**	8.7 ± 2.2	7.9 ± 1.98	-0.8	< 0.001	8.2 ± 2.3	7.7 ± 1.8	-2.3	0.01	0.66	0.66
**Insulin (mIU/L)**	7.5 ± 2.4	7.5 ± 2.59	0.7	0.94	7.9 ± 2.3	5.8 ± 2.4	-3.5	0.01	0.08	0.08
**HOMA-IR**	3.4 ± 2.5	2.7 ± 2.63	-0.6	0.19	3.5 ± 2.4	2.1 ± 2.3	-30.5	0.001	0.08	0.08
**QUICKI**	0.3 ± 0.1	0.3 ± 0.05	0.0	0.24	0.3 ± 0.0	0.3 ± 0.0	-0.9	0.001	0.25	0.27
**SBP (mm Hg)**	127 ± 13.8	108 ± 35.8	-18.3	0.004	128.8 ± 17.6	110.2 ± 43.2	-3.5	0.01	0.99	0.99
**DBP (mm Hg)**	77 ± 6.3	70 ± 20.1	-6.5	< 0.05	79.4 ± 11.6	80.0 ± 11.6	0.6	0.78	0.05	0.05
**TC (mm Hg)**	187.9 ± 50.1	183.0 ± 47.3	-4.9	0.50	178.7 ± 45.7	180.2 ± 49.5	1.5	0.69	0.55	0.57
**HDL (mg/dl)**	41.7 ± 7.7	50.2 ± 12.5	8.5	0.002	40.4 ± 9.9	49.3 ± 12.0	8.9	0.005	0.753	0.753
**LDL (mg/dl)**	98.3 ± 31.8	95.0 ± 25.7	-3.3	0.42	96.2 ± 33.7	90.8 ± 28.89	-5.45	0.014	0.481	0.479
**TG (mg/dl)**	163.1 ± 1.6	148.5 ± 1.6	-13.7	< 0.05	147.1 ± 1.7	127.58 ± 1.68	-24.93	0.003	0.303	0.302

*BMI* Body mass index, *FBG* Fasting blood glucose, *HbA1C* Hemoglobin A1C, *SBP Systolic blood pressure*, *DBP* Diastolic blood pressure, *TC* Total cholesterol*, HDL* High-density lipoprotein, *LDL* Low-density lipoprotein*, TG* Triglyceride, *HOMA-IR* Homeostatic model assessment for insulin resistance, *QUICKI* Quantitative insulin sensitivity check inde

^a^Variables are expressed as mean ± SD except for insulin, HOMA-IR and TG reported as geometric mean ± SD

^b^Comparison between baseline and endpoint, obtained from Paired T test

^c^Obtained from ANCOVA, adjusted for baseline value

^d^Obtained from ANCOVA, adjusted for baseline value after intention to treat

### Primary outcomes

Blood glucose indices showed that the changes in FBG and HbA1C were significantly changedwithin both groups. Insulin, HOMA-IR, QUICKI and LDL were significantly decreased compared with baseline in the control group. Analysis were repeated after using ITT method (Table 3). Nevertheless, similar findins were observed.

### Secondary outcomes

Weight, BMI,, SBP, HDL and TG were significantly changed within both groups. In the LRALD group, DBP was significantly decreased after intervention. LDL was significantly decreased compared with baseline in the control group. After adjusting for baseline measurements, the comparison of the final values in the two groups showed that a LRALD marginally decreased DBP compared with the control diet (Table 3). Similar findings were observed after using ITT method (Table 3).

## Discussion

In the present study, two dietary interventions were compared in type 2 diabetic patients. The most important innovation of the present study was the utilization of dietary acid load as a nutritional intervention. a finding of the present study was a significant reduction in DBP after adherence to a LRALD compared with control intervention. Also, SBP was significantly reduced after following a LRALD compared with the beginning of the study. While, in the control group, only a decrease in SBP was observed. Indeed, these findings indicated that a LRALD had more beneficial effects than a usual diabetic diet on blood pressure in patients with type 2 diabetes. The prevalence of hypertension in patients with type 2 diabetes is notably prevalent compared with healthy individuals [29]; indeed, most type 2 diabetic patients have a high blood pressure at type 2 diabetes diagnosis. Also, there is a direct relationship between blood pressure and risk of nephropathy, retinopathy, neuropathy, and cardiovascular disease in patients with type 2 diabetes [30]; therefore, it is plausible that a LRALD could play an important role in reducing the side effects of type 2 diabetes by lowering blood pressure.

A possible mechanism of the decreasing impact of a LRALD on blood pressure is the presence of abundant phenolic compounds in plants found in high amounts in this diet [31]. The hypothesis of the effect of phenolic compounds on blood pressure has various mechanisms, including the effect of phenolic acids on NO-mediated vasodilatory response of endothelial wall, decreasing oxidative stress by reduction in NAD (P) H-dependent (nicotinamide adenine dinucleotide phosphate) super oxide products, and inhibiting the activity of the angiotensin-converting enzyme [32]. It has previously been observed that a diet with a high acid load can induce the glutaminase enzyme and activate the renin-angiotensin system, leading to an increase in blood pressure [33]. Moreover, blood uric acid is directly related to high blood pressure and a diet with low acid content can lead to an increase in urinary uric acid clearance and a decrease in blood uric acid [34].

Previous studies have reported findings similar to results of the present study regarding the effect of a LRALD on blood glucose indices. A cross-sectional study in Japan found that there was no correlation between dietary acid load, FBG and HBA1c [35]. Indeed, these results are consistent with the findings of the present study.

The findings of the current study regarding the effect of an LRALD on blood pressure has been confirmed by previous studies. A cross-sectional study reported that PRAL and NEAP were positively associated with DBP in men and SBP in women [36], whilst a meta-analysis that pooled the results of the 14 studies reported that there was a positive association between dietary acid load and blood pressure. In this meta-analysis, it was revealed that each 20 units increase in PRAL elevated the risk of hypertension by 3%, also, a significant nonlinear relationship was found between NEAP and blood pressure [37].

The present study showed a no significant effect of LRALD on lipid profile, similar to a previous study which found no relationship between NEAP and HDL, LDL, and TC [17]. It should be acknowledged that the concentration of LDL, TC, HDL, and TG was in normal range at baseline, and it is unlikely that dietary interventions can change blood lipids within normal physiological range. Nevertheless, some studies have reported that other healthy diets similar to LRALD, such as DASH diet, failed to change lipid profile in normal range [38].

Three dietary patterns, including LRALD, DASH, and Mediterranean diet, have numerous similarities; for example, high-fat cheeses, red meat, and egg yolks are limited in all three diets. Instead of red meat, the consumption of legumes and chicken and fish in DASH and LRALD is recommended, also, olive oil is recommended in these three dietary patterns. Sodium restriction is also encouraged in all three diets [39]. Therefore, it is suggested that future research examine the effects of combined dietary patterns, such as the low renal acid load DASH diet or the low renal acid load Mediterranean diet, to provide patients with all the benefits of these diets.

Although we have provided a novel addition to literature, which may be a practical relevance to prescribing clinicians and patients, there are limitations that should be acknowledged. One of the limitations of this study is the lack of examining of individuals' compliance with the intervention via biomarker. Assessment of compliance with the LRALD requires measuring urine pH over 24 h, however, collecting 24-h urine sample is difficult and it may affect the reliability of the results [40]. Therefore, multiple food diaries were used to assess compliance in the present study. Another limitation of the present study was the loss of some participants due to the prevalence of COVID-19 and lockdown. Nevertheless, it should be noted that the number of subjects who completed the study was more than minimum required sample size. Social stress in people during the COVID-19 outbreak can also be one of the co-variants that leads to impaired blood glucose regulation in type 2 diabetic patients, and it may have a negative effect on the results.

## Conclusion

This study showed that a LRALD had beneficial effects on blood pressure in type 2 diabetic patients compared with the usual diabetic diet, but its effect on blood glucose control factors and lipid profile was not significant.

## Supplementary Information

**Additional file 1: Supplementary 1.** Dietary recommendations for control and LRAL group.

**Additional file 2: Supplementary 2.** Potential renal acid load score of foods.

## Data Availability

Data will be available on request.
