# Supplementary material for: The effect of a low renal acid load diet on blood pressure, lipid profile, and blood glucose indices in patients with type 2 diabetes: a randomized clinical trial
Source: Nutr J. 2023 Mar 15;22:18. doi: 10.1186/s12937-023-00849-6 (PMC10014397; doi:10.1186/s12937-023-00849-6)
Supplement: Supplementary file 1 — Additional file 1: Supplementary 1. Dietary recommendations for control and LRAL group. [file 12937_2023_849_MOESM1_ESM.docx]

**Supplementary 1**. Dietary recommendations for control and LRAL group

| **Common dietary recommendations for both intervention and control groups** | **Dietary Measures and Recommendations Specifically for the intervention group (LRAL DIET(** |
| --- | --- |
| Daily meal plans were designed according to the diabetic patients (Such as carbohydrate counting ( | Foods with high acid load (PRAL>4), except chicken meat, were excluded from the diet of LRALD group |
| Meals should be small, frequent and used regularly | Chicken meat, one of the most frequently consumed foods, was limited to one serving per day |
| Do not change carbohydrate content of your diet without consulting your dietician | two fixed snacks contained very low PRAL foods (foods with PRAL< -4 such as spinach, celery, squash, and raisins) were prescribed in LRALD group |
| Restrict intake of refined carbohydrate, Whole grains are preferable to refined grains | A colored list of the food items was provided, in which the red color was used for foods with high PRAL and low-PRAL foods were demarcated by the color green. Subjects in the LRALD group were educated to select green items and limit red foods |
| Eat vegetables frequently |  |
| Use fruits with skin if possible |  |
| Fruits are preferable to fruit juice |  |
