# Supplementary material for: The effect of a low renal acid load diet on blood pressure, lipid profile, and blood glucose indices in patients with type 2 diabetes: a randomized clinical trial
Source: Nutr J. 2023 Mar 15;22:18. doi: 10.1186/s12937-023-00849-6 (PMC10014397; doi:10.1186/s12937-023-00849-6)
Supplement: Supplementary file 2 — Additional file 2: Supplementary 2. Potential renal acid load score of foods. [file 12937_2023_849_MOESM2_ESM.docx]

**Supplementary 2**. Potential renal acid load score of foods

| **Food item** | **Potential renal acid load (PRAL)** |
| --- | --- |
| **Raisins** | -21 |
| **Spinach** | -14 |
| **Black currants** | -6.5 |
| **Celery** | -5.2 |
| **Carrots, young** | -4.9 |
| **Apricots** | -4.8 |
| **Zucchini** | -4.6 |
| **Kiwi fruit** | -4.1 |
| **Cauliflower** | -4 |
| **Radish, red** | -3.7 |
| **Eggplant** | -3.4 |
| **Tomatoes** | -3.1 |
| **Beans, green/French beans** | -3.1 |
| **Pears,3 varieties, flesh and skin, average** | -2.9 |
| **Orange juice, unsweetened** | -2.9 |
| **Tomato juice** | -2.8 |
| **Hazelnut’s** | -2.8 |
| **Pineapple** | -2.7 |
| **Oranges** | -2.7 |
| **Lettuce, average of varieties** | -2.5 |
| **Lemon juice** | -2.- |
| **Peaches** | -2.4 |
| **Red wine** | -2.4 |
| **Strawberries** | -2.2 |
| **Apples,15 varieties, flesh and skin, average** | -2.2 |
| **Apple juice, unsweetened** | -2.2 |
| **Chicory** | -2 |
| **watermelon** | -1.9 |
| **Mineral water (apollinaris)** | -1.8 |
| **Coffe,infusion,5minutes** | -1.4 |
| **White wine, dry** | -1.2 |
| **Grape juice, unsweetened** | -1 |
| **Margarine** | -0.5 |
| **Cocoa, made with semi-skimmed milk** | -0.4 |
| **Tea, indian, infusion** | -0.3 |
| **Beer, draft** | -0.2 |
| **Beer, stout, bottled** | -0.1 |
| **Olive oil** | 0 |
| **Sunflower seed oil** | 0 |
| **Mineral water (volvic)** | 0.1 |
| **Coca -cola** | 0.4 |
| **Buttermilk** | 0.5 |
| **Butter** | 0.6 |
| **Ice cream, dairy, vanilla** | 0.6 |
| **Milk, whole, pasteurized and sterilized** | 0.7 |
| **Beer, pale (vollbier. hell)** | 0.9 |
| **Eggs, white** | 1.1 |
| **Milk, whole, evaporated** | 1.1 |
| **Yogurt, whole milk, fruit** | 1.2 |
| **Creams, fresh, sour** | 1.2 |
| **Peas** | 1.2 |
| **Yogurt, whole milk, plain** | 1.5 |
| **Rice. white, easy cook, boiled** | 1.7 |
| **Bread, wheat flour, whole meal** | 1.8 |
| **Chocolates, milk** | 2.4 |
| **Crispbread, rye** | 3.3 |
| **Lentils, green and brown, whole, dried** | 3.5 |
| **Cherries** | 3.6 |
| **Madeira cake** | 3.7 |
| **Bread ,white wheat** | 3.7 |
| **Bread, wheat flour, mixed** | 3.8 |
| **Bread, rye flour, mixed** | 4 |
| **Bread, rye flour** | 4.1 |
| **Full-fat soft cheese** | 4.3 |
| **Rice, white, easy cook** | 4.6 |
| **Bananas** | 5.5 |
| **Rye flour, whole** | 5.9 |
| **Cornflakes** | 6 |
| **Noodles, egg** | 6.4 |
| **Spaghetti, white** | 6.5 |
| **Frankfurters** | 6.7 |
| **Walnuts** | 6.8 |
| **Haddock** | 6.8 |
| **Wheat flour, white, plain** | 6.9 |
| **Herring** | 7 |
| **Cod fillets** | 7.1 |
| **Spaghetti, whole meal** | 7.3 |
| **Beef, lean only** | 7.8 |
| **Pork, lean only** | 7.9 |
| **White flour, whole meal** | 8.2 |
| **Peanuts, plain** | 8.3 |
| **Chicken, meat only** | 8.7 |
| **Cottage cheese, plain** | 8.7 |
| **Rump steak, lean and fat** | 8.8 |
| **Veal, fillet** | 9 |
| **Turkey, meat only** | 9.9 |
| **Luncheon meat, canned** | 10.2 |
| **Liver sausage** | 10.6 |
| **Oat flakes, rolled oats(Haferflocken)** | 10.7 |
| **Trout, brown, steamed** | 10.8 |
| **Fresh cheese(quark)** | 11.1 |
| **Salami** | 11.6 |
| **Rice, brown** | 12.5 |
| **Corned beef ,canned** | 13.2 |
| **Camembert** | 14.6 |
| **Cheese gouda** | 18.6 |
| **Hard cheese, average of 4 types** | 19.2 |
| **Eggs, yolk** | 23.4 |
| **Cheddar-type, reduced fat** | 26.4 |
| **Processed cheese, plain** | 28.7 |
| **parmesan** | 34.2 |
